# Neonatal diabetes and protein losing enteropathy: a case report

**DOI:** 10.1186/s12881-016-0296-0

**Published:** 2016-04-21

**Authors:** Tamara McMillan, Rose Girgis, Elizabeth A. C. Sellers

**Affiliations:** Department of Paediatrics and Child Health, University of Manitoba, FE 307-685 William Avenue, Winnipeg, Manitoba R3E 0Z2 Canada; Department of Paediatrics, University of Alberta, Alberta, Canada

**Keywords:** Neonatal diabetes, pancreatic hypoplasia, protein losing enteropathy

## Abstract

**Background:**

Neonatal diabetes is a rare form of monogenic diabetes with onset in the first six months of life occurring in 1/100,000 to 1/400,000 births. Both permanent and transient forms have been described. Permanent neonatal diabetes results predominantly from mutations in the KCNJ11 and ABCC8 genes. Less frequently, mutations of the GATA6 gene, located on chromosome 18 cause a form of permanent neonatal diabetes resulting from pancreatic hypoplasia or agenesis. Other anomalies associated with mutations of this gene have also been reported, most commonly congenital heart disease.

**Case presentation:**

We report the case of a Caucasian male infant diagnosed shortly after birth with neonatal diabetes, truncus arteriosus type III, ventricular septal defect, atrial septal defect, an absent gallbladder and a right inguinal hernia. His diabetes resulted from a de novo mutation of the GATA6 gene resulting in pancreatic hypoplasia. At 20 months of age he developed protein losing enteropathy. This has not previously been associated with GATA6 mutations and it is not known if this association is causal.

**Conclusion:**

The combination of neonatal diabetes and pancreatic agenesis/hypoplasia should alert the clinician to the possibility of a GATA6 gene abnormality. The association of protein losing enteropathy is unique to the reported case.

## Background

Neonatal diabetes is a monogenic form of diabetes with onset in the first 6 months of life. It occurs in 1 in 100,000 to 400,000 births [1]. Transient neonatal diabetes resolves within the first 18 months and predisposes to the development of diabetes later in life [2]. The most common underlying genetic causes of transient neonatal diabetes are due to imprinting defects of chromosome 6 [2]. Permanent neonatal diabetes is predominantly due to mutations in the KCNJ11 and ABCC8 genes which encode for subunits of the sulfonylurea receptor. Permanent neonatal diabetes can also be caused by mutations in the insulin gene or the GCK gene which encodes for the glucokinase enzyme [2].

Less commonly, neonatal diabetes results from pancreatic hypoplasia or agenesis. In a large study of 795 patients with neonatal diabetes, 39/795 (4.9 %) were found to have pancreatic agenesis (defined by the need for both insulin and having pancreatic exocrine insufficiency and need for enzyme replacement therapy)[3]. Mutations of PRX1 and PTF1A (found on chromosomes 13 and 10 respectively) are associated with pancreatic hypoplasia/agenesis. These genes encode for transcription factors required for pancreatic growth and development [4]. The GATA6 gene, on chromosome 18, has also been linked to neonatal diabetes and is the most common cause of pancreatic agenesis accounting for just over 50 % of cases [3]. This gene encodes for GATA6, a transcription factor involved in the development of multiple organ systems including the pancreas. Mutations of the GATA 6 gene may result in pancreatic hypoplasia or agenesis and are associated with other anomalies, most commonly congenital heart disease [5]. GATA6 mutations account for approx. 3 % of all cases of neonatal diabetes [3].

We report an infant presenting with neonatal diabetes resulting from a de novo heterozygous mutation in the GATA6 gene.

Submission of this report was approved by the Human Research Ethics Board, Faculty of Health Sciences, University of Manitoba.

## Case presentation

A Caucasian male infant was born to a 33 year old G7P5SA2 mother and her non-consanguineous partner at 37 weeks gestation. The pregnancy was complicated by morning sickness treated with Diclectin® (doxylamine succinate/pyridoxine hydrochloride). There were no other exposures to known teratogens. Intrauterine growth restriction was noted on ultrasound at 20 weeks gestation (head circumference, weight, femur length all < 5%ile). Oligohydramnios was noted at 34 weeks gestation, and the mother was placed on bed rest.

Labour was induced at 37 weeks gestation for asymmetric growth restriction. The infant was delivered by caesarean section for failure to progress. Birth weight was 1580 grams (<3rd %ile), length was 40 centimeters (<3rd %ile) and head circumference was 31 centimeters (3rd-10th %ile). Apgar scores were 9 and 9 at 1 and 5 minutes. Newborn examination revealed a small, non-dysmorphic baby with minimal body fat, a heart murmur and a right sided inguinal hernia.

Hyperglycemia was noted at 12 hours of age and an insulin infusion was started. An echocardiogram identified truncus arteriosus type III, hypoplastic pulmonary arteries, a ventricular septal defect and atrial septal defect (VSD and ASD respectively). He underwent cardiac repair on day 7 of life. Surgical repair involved the construction of a right ventricular – pulmonary artery conduit, patch closure of the VSD and suture closure of the ASD.

The pancreas was not identified on ultrasound. Abdominal magnetic resonance imaging (MRI) demonstrated minimal ectopic pancreatic tissue, absent gall bladder, liver steatosis and normal kidneys and spleen. Cerebral MRI was normal. C-peptide levels were undetectable, amylase and lipase were low and fecal fat was elevated suggesting pancreatic endocrine and exocrine deficiencies. Pancreatic exocrine replacement therapy was started week 3 of life (Creon® pancrelipase delayed release capsules 2500 u lipase/kg/d).

Family history revealed no history of cardiac or other congenital abnormalities. The mother is known to be a carrier of a balanced Robertsonian translocation (45, XX der(13;14)(q11;q10). She had had two early miscarriages, thought to be related to an unbalanced translocation. The maternal aunt and grandfather have type 2 diabetes.

Our patient was found to carry the same balanced Robertsonian translocation as his mother. Microarray testing was normal. Based on the combination of a congenital heart defect and pancreatic agenesis, GATA6 gene mutation testing was performed. A frameshift deletion in exon 2 (c744del) was found predicting a premature stop codon. Neither parent carries this mutation.

At four months of age the infant was transitioned from a continuous insulin infusion to subcutaneous injections. His course has been complicated by congenital hip dysplasia, developmental delay, and recurrent episodes of bronchiolitis. At 22 months of age, the child presented with facial edema, abdominal distension and poor feeding. Serum albumin was 20 g/L (normal range 35-47 g/L ). Subsequent investigations revealed a normal urinalysis and normal urine albumin. Testing for celiac disease was negative. Upper endoscopy with video capsule placement demonstrated diffuse erythema and superficial ulceration of the stomach and edema throughout the small bowel. Small bowel lymphangiectasia was also noted. Sigmoidoscopy to 20 cm did not demonstrate any abnormalities of the colon. These findings lead to the diagnosis of protein losing enteropathy. Treatment with enoxaparin and oral budesonide was initiated with good clinical response.

At 30 months of age he weighs 11.59 kilograms (15th %ile) and he has a height of 80 centimeters (<3rd %ile). His diabetes is managed on a basal-bolus regime with insulin glargine and lispro (diluted) for carbohydrate intake and correction. HbA1c is 7.4 % (normal 4-6 %). Average total daily dose is 0.15 - 0.2 units/kg/day. His low insulin requirements are thought to be due to endogenous production of insulin by ectopic pancreatic tissue identified on MRI. Fortunately, the child has not experienced any episodes of severe hypoglycemia.

## Conclusions

We report an infant presenting with neonatal diabetes associated with congenital heart disease, absent gall bladder and an inguinal hernia resulting from a de novo heterozygous mutation in the GATA6 gene. The infant has a number of associated anomalies including the unique description of protein losing enteropathy.

In the largest published series of patients with a GATA6 mutation, 24 probands and 5 parents were characterised [3]. All but one patient, who was a mosaic mutation carrier, developed diabetes. The majority had permanent neonatal diabetes, but one had transient episodes of hyperglycemia and four developed diabetes between the ages of 12 and 46 years. In this same cohort, 93 % had evidence of high fecal fat or required pancreatic enzyme supplementation, 83 % had cardiac malformations, 24 % had hypothyroidism or hypopituitarism and 38 % had neurocognitive deficits.

To the best of our knowledge, this is the first report of a patient with a GATA6 mutation with pancreatic agenesis and congenital heart disease complicated by protein losing enteropathy. Protein losing enteropathy is often related to congenital heart defects associated with increased right-sided pressures; however, our patient had normal right sided heart pressures. If his protein losing enteropathy is attributed to his heart defect, to our knowledge, he is the first reported case secondary to truncus arteriousus. The GATA6 gene is known to be involved in intestinal development [6]. Hepatobiliary malformations and gut abnormalities (malrotation and hernias) have been reported [3, 5]. This suggests the possibility that the protein losing enteropathy in our patient is related to his gene mutation.

We report a case of neonatal diabetes resulting from a de novo mutation of the GATA6 gene resulting in pancreatic hypoplasia. Our case has several associated anomalies including truncus arteriosus, gallbladder agenesis, an inguinal hernia and uniquely, protein losing enteropathy illustrating the variable phenotype associated with mutations of this gene. Although uncommon, it is important to consider GATA6 mutations and investigate for pancreatic agenesis/hypoplasia if neonatal diabetes is seen in combination with congenital heart defects or other anomalies. Given the variability in phenotype, parents should be screened for carrier status. Carriers may benefit from early screening for diabetes as age of presentation varies significantly. The combination of neonatal diabetes and other congenital anomalies should alert the clinician to the possibility of a GATA6 gene abnormality.

### Ethics

Submission of this report was approved by the Human Research Ethics Board, Faculty of Health Sciences, University of Manitoba. Written informed consent was obtained from the patient’s guardian for publication of this Case report. A copy of the written consent is available for review by the Editor of this journal.

### Availability of data and materials

All data supporting our findings are included in the manuscript.

## Abbreviations

ASD: atrial septal defect
MRI: magnetic resonance imaging
VSD: ventricular septal defect
